# Endothelial dysfunction and metabolic biomarkers in post-COVID-19 syndrome

**DOI:** 10.1038/s41598-026-50965-6

**Published:** 2026-05-13

**Authors:** Martin Oestreich, Maria Schmidt, Julia Dittrich, Ronny Baber, Alexander Gaudl, Madlen Reinicke, Anja Willenberg, Kerstin Wirkner, Florian Then Bergh, Christopher Fricke, Alexandra Rockstroh, Jasmin Fertey, Sebastian Ulbert, Jörg Lehmann, Claudia Müller, Milica Matijevic, Samira Zeynalova, Christoph Engel, Markus Löffler, Berend Isermann, Uta Ceglarek, Ronald Biemann

**Affiliations:** 1 Institute of Laboratory Medicine, Clinical Chemistry and Molecular Diagnostics, University of Leipzig, Paul-List- Straße 13-15, 04103 Leipzig, Germany; 2 Institute for Medical Informatics, Statistics and Epidemiology, University of Leipzig, 04107 Leipzig, Germany; 3 Leipzig Research Centre for Civilization Diseases, University of Leipzig, 04103 Leipzig, Germany; 4 Department of Neurology, University of Leipzig, 04103 Leipzig, Germany; 5 Department of Preclinical Development and Validation, Fraunhofer Institute for Cell Therapy and Immunology – IZI, 04103 Leipzig, Germany; 6 Leipzig Medical Biobank, University of Leipzig, 04103 Leipzig, Germany

**Keywords:** COVID-19, Post-COVID-19 syndrome, Endothelial dysfunction, MFI-20, Biomarkers, Diseases, Medical research

## Abstract

Acute SARS-CoV-2 infection has been implicated in the development of endothelial dysfunction and metabolic alterations. These disturbances may contribute to the pathophysiology of post-COVID-19 syndrome (PCS), a multifaceted condition characterized by persistent symptoms, including neuropsychiatric symptoms. The diagnosis of PCS primarily relies on symptom-based criteria. Here, we aimed to identify biomarkers associated with PCS and disease severity. This prospective single-center cohort study investigated soluble blood biomarkers related to endothelial dysfunction and amino acid, fatty acid, carnitine, eicosanoid and resolvin metabolism in individuals post-acute SARS-CoV-2 infection with or without PCS compared with individuals without documented SARS-CoV-2 infection. Additionally, we explored the association between these biomarkers and PCS-related fatigue severity as assessed by the Multidimensional Fatigue Inventory (MFI). At a median of 37.4 weeks after SARS-CoV-2 infection, participants with prior infection showed higher levels of soluble thrombomodulin (TM) and L-lactate dehydrogenase (LDH) than those without previous infection. Alterations in arginine biosynthesis and taurine and hypotaurine metabolism indicate disruption of the NO-metabolism. These findings were made in participants without and with symptoms of PCS. In participants with PCS-related high fatigue severity, concentrations of the polyunsaturated fatty acid (PUFA) linoleic acid (LA), and the monounsaturated fatty acids (MUFAs) oleic acid (OA) and palmitoleic acid (PA) were higher than in participants with low fatigue severity. Alterations in markers of endothelial dysfunction and NO-metabolism are detectable at a median of 37.4 weeks after SARS-CoV-2 infection independent of PCS-related fatigue severity. Additionally, in individuals with high PCS-related fatigue severity, specific fatty acid alterations were observed.

**Supplementary Information:**

The online version contains supplementary material available at 10.1038/s41598-026-50965-6.

## Introduction

According to the World Health Organization (WHO), the post-COVID-19 syndrome (PCS) is a condition characterized by symptoms that persist for at least three months following infection with SARS-CoV-2, last for more than two additional months, and cannot be attributed to any other underlying disease^1^. In contrast, the term “long COVID” refers to symptoms that persist or develop in adults between 4 and 12 weeks following infection, but do not extend beyond this period.

A hallmark of PCS is a persistent inflammatory response: elevated levels of inflammatory markers, including C-reactive protein, D-dimer and leukocytes have been observed in patients after COVID-19 infection with PCS compared with those without prolonged symptoms^2^, suggesting that chronic inflammation plays a role in PCS pathophysiology and may act through endothelial dysfunction (ED). Amino acid metabolism plays a crucial role in inflammation, for example with tryptophan metabolites influencing anti-inflammatory pathways^3^. Acylcarnitines contribute to inflammatory processes by activating pro-inflammatory signaling and affecting fatty acid metabolism in immune cells^4,5^. Mounting evidence highlights relevant metabolic dysregulation in PCS, with enduring disruptions in energy metabolism, amino acid metabolism, and lipid metabolism persisting for up to two years post-infection^6^.

Inflammation is frequently associated with ED. Additionally, ED is closely linked to altered amino acid metabolism, particularly through the role of L-arginine, homocysteine, and other amino acids involved in nitric oxide (NO) biosynthesis, a key factor in maintaining endothelial function^7,8^. Persistent ED has emerged as a pivotal link between acute COVID-19 and the chronic symptoms observed in PCS^9^.

Metabolic disturbances and immune dysregulation are increasingly recognized as critical pathophysiological contributors to hallmarks of chronic symptoms such as fatigue and neuropsychiatric symptoms^10^. Fatigue, often described as a debilitating and persistent state of exhaustion, is one of the most commonly reported symptoms among individuals with PCS and individuals recovering from COVID-19. A systematic review and meta-analysis highlights that fatigue is prevalent in approximately 32% of individuals 12 or more weeks following a COVID-19 diagnosis, indicating a substantial burden on their quality of life^11^. In PCS, fatigue is the most commonly reported symptom, with an 85% prevalence rate^12^. Underlying mechanisms explaining fatigue severity in PCS are not fully understood and are suggested to be multifactorial^13^. Collectively, chronic inflammation, ED, and metabolic disturbances have emerged as key features of PCS^2,9,10^ and may also drive PCS-related fatigue severity.

The current body of research on the observed changes in long- and post-COVID syndrome in non-hospitalized individuals is primarily based on studies with relatively small sample sizes, often lacking non-infected controls. The aim of the current study was to compare soluble blood biomarkers of ED and amino acid, fatty acid, carnitine, eicosanoid and resolvin related metabolism in a well-characterized cohort of individuals with or without PCS at a median of 37.4 weeks post-acute infection and to investigate whether these biomarkers are associated with PCS-related fatigue severity.

## Materials and methods

### Recruitment and participants

**Fig. 1:**
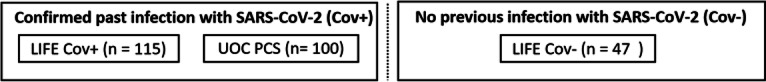
Study scheme to investigate the influence of previous infection with SARS-CoV-2 on biomarkers for ED and amino acid, fatty acid, carnitine, eicosanoid and resolvin related metabolism. 115 study participants of the LIFE study (LIFE Cov+) and 100 patients from the university outpatient clinic (UOC PCS) fulfill the inclusion and exclusion criteria for subjects with previous infection with SARS-CoV-2. 47 study participants of the LIFE study whithout previous SARS-CoV-2 infection were included in the control group (LIFE Cov-). LIFE Cov-: Leipzig Research Center for Civilization Diseases, Covid negative; LIFE Cov+: Leipzig Research Center for Civilization Diseases, Covid positive; UOC PCS: university outpatient clinic with post-COVID syndrome.

This prospective single-center cohort study aimed to investigate the long-term health impacts of SARS-CoV-2 infection, focusing on clinical, laboratory, and self-reported data. Between July 2021 and January 2022, 100 adults with a previous SARS-CoV-2-infection were recruited via the University outpatient post-COVID clinic at University of Leipzig Medical Center (UOC PCS), many of whom reported persistent health complaints after documented SARS-CoV-2 infection with a median time from last SARS-CoV-2 infection to consultation of 36.1 weeks. In addition, 115 participants with previous SARS-CoV-2-infection were recruited from among the LIFE-Adult Study (a population-based study cohort from Leipzig, Germany), between August 2021 and January 2022 (LIFE Cov+), with a median duration from most recent SARS-CoV-2 infection to consultation of 38.8 weeks. Further 47 participants from the LIFE-Adult-Study without a previous SARS-CoV-2-infection (LIFE Cov-) were included as a control group (Fig. 1) between December 2021, and January 2022. The LIFE-Adult-Study is described in detail elsewhere^14,15^. Inclusion criteria for participants of the UOC PCS cohort were the ability to visit the UOC, presentation at least 3 months after the acute course of SARS-CoV-2-infection, infection confirmed by SARS-CoV-2 polymerase chain reaction (PCR) test, and at least one persisting symptom 12 weeks after acute infection^16^. Participants were included in the LIFE Cov+ cohort if they participated in the LIFE-Adult study and had a self-reported history of SARS-CoV-2 infection. Participants in the UOC PCS cohort attended the outpatient clinic due to subjective persistent symptoms, whereas inclusion in the LIFE Cov+ cohort was based on a history of SARS-CoV-2 infection, irrespective of symptom status. Data on the period since SARS-CoV-2 infection were summarized for LIFE Cov+ (38.8 weeks) and UOC PCS (36.1 weeks) to 37.4 weeks in both groups, the date was missing for 1 participant in the UOC PCS cohort and for 13 participants in the LIFE Cov+ cohort. 10 out of 272 participants were excluded from the study due to missing information (i.e., missing laboratory parameters, smoking status and fatty acid data).

### Laboratory assessments

Venous blood was collected on the day of the participant’s visit and centrifuged within 30–45 min after blood collection (except for blood counts). Aliquots of serum, EDTA- and citrated plasma were frozen either in the gas phase of liquid nitrogen or at − 80 °C by the Leipzig Medical Biobank following standard operating procedures until further use. Supplementary Table 2 provides an overview of the laboratory analyses. Leukocytes, glucose, alanine aminotransferase, aspartate aminotransferase, high-density lipoprotein, small dense low-densitiy lipoprotein, high-sensitivity CRP, lactate dehydrogenase, prothrombin time, activated partial thromboplastin time, fibrinogen, D-dimer, protein C antigen and von Willebrand factor antigen were analysed at the Institute of Laboratory Medicine, Clinical Chemistry and Molecular Diagnostics (University of Leipzig Medical Center, Germany) according to the accreditation standards DIN EN ISO 15,189. Soluble Thrombomodulin (TM), soluble intercellular adhesion molecule-1 (ICAM-1), and soluble vascular cell adhesion molecule-1 (VCAM-1) were quantified using enzyme-linked immunosorbent assays (ELISA) according to the manufacturer’s instructions (TM: DTHBD0, ICAM-1: DCD540, VCAM-1, DVC00; R&D Systems, Inc., Minneapolis, USA). Targeted LC-MS/MS analysis for quantification of PUFAs, MUFAs and eicosanoids including resolvins was performed as described previously^17,18^. Amino acid and acylcarnitine metabolite quantification was performed according to our previously described methods^19^.

### Self-report questionnaires

All participants underwent structured interviews at the LIFE Adult study center, including interviews and questionnaires regarding personal health conditions, cardiopulmonary symptoms and medication use. Anthropometric measurements, such as height, weight, and body mass index, as well as blood pressure assessments were performed by trained staff. The Multidimensional Fatigue Inventory (MFI- 20;^20^ was used to assess fatigue-related PCS symptom load. The questionnaire includes five subscales: general fatigue, physical fatigue, reduced activity, reduced motivation and mental fatigue.

### Statistical analysis

Statistical analyses were performed using R version 4.2.1 (http://www.R-project.org). Continuous data are presented as medians with interquartile ranges (IQR), and categorical data are shown as absolute numbers and percentages. Group differences in continuous variables were assessed using the Mann-Whitney U test or the Kruskal-Wallis test with post hoc Dunn tests, while differences in categorical variables were analysed using Fisher’s exact test or the Fisher-Freeman-Halton test with pairwise Fisher tests. Median imputation was applied to address missing values in biomarkers related to ED. Biomarkers related to fatty acid, eicosanoid, and resolvin metabolism that were below the lower limit of quantification (LLOQ) or undetected were coded as 0 if the proportion of these values was ≤ 5%; biomarkers with more than 5% of these values were excluded from the analysis. Biomarkers were log transformed to achieve normal distributions, and then z-scaled. Success of transformations was visually assessed using qq-plots. Using linear regression, we further investigate the effect of the variable „group“ on each of the 94 metabolites, including markers of ED and amino acid, fatty acid, eicosanoid and resolvin related metabolism while adjusting for potential confounding variables. The false discovery rate (FDR) within metabolite groups was subsequently controlled for using Benjamini-Hochberg correction^21^. Correlation between biomarkers was assessed using Spearman correlation, focusing on correlations >|0.3|. Pathway analysis was performed using MetaboAnalyst 6.0^22^ with the Kyoto Encyclopedia of Genes and Genomes (KEGG) pathway library for Homo sapiens (retrieved December 2023). The pathway analysis employed the Hypergeometric Test to identify significantly impacted pathways by comparing detected metabolites to the reference metabolome, while Relative Betweenness Centrality was used to assess pathway topology^23^. Results were visualized in a scatter plot, highlighting pathways’ impact scores and statistical significance (*p* < 0.05).

For statistical and graphical tasks, the following R packages were utilized: ggplot2^24^; reshape2^25^ RColorBrewer^26^; corrplot^27^; dplyr^28^. dunn.test^29^; rcompanion^30^: ggpubr^31^; cowplot^32^ and ggrepel^33^.

### Ethics approval and consent to participate

The study is conducted in accordance with the Declaration of Helsinki and was approved by the responsible ethics board at the Medical Faculty of the University of Leipzig (reference: 345/21-ek). All participants provided written informed consent to participate prior to participation.

## Results

Blood biomarkers were investigated in participants from the University Outpatient post-COVID Clinic (UOC PCS) and from the LIFE Adult study (LIFE Cov+) at a median of 37.4 weeks post-infection and compared with respective analytes in controls without previous infection with SARS-CoV-2 (LIFE Cov-) as described in Fig. 1.

Baseline comparison of subjects with previous SARS-CoV-2 infection (UOC PCS and LIFE Cov+) and those without (LIFE Cov-) showed differences in age, frequency of smoking, hypertension and depression (Table 1). The cohorts were described previously^34^. It is important to note that participants of the UOC PCS cohort (median 45 years) and the LIFE Cov- cohort (median 50 years) were younger compared with the LIFE Cov+ cohort (median 58 years). Additionally, current smoking status was less common in the UOC PCS cohort (9.0%) and the LIFE Cov+ cohort (7.8%) compared with the LIFE Cov- cohort (25.5%). To account for these disparities, adjustments for age, current smoking status and hypertension were incorporated in the linear regression analysis (Table 1).

**Table 1:** Baseline characteristics of the study population consisting of participants with previous SARS-CoV-2 infections (Cov+, *n* = 215) including LIFE Cov+ (*n* = 115) and UOC PCS (*n* = 100) and controls without previous infection with SARS-CoV-2 (Cov-, *n* = 47). Differences between the groups were analysed using the Kruskal-Wallis test for continuous variables followed by Dunn’s test for post-hoc pairwise comparisons. Fisher’s exact test was used to analyze categorical variables, and pairwise Fisher tests were conducted as post hoc tests. Continuous variables are presented as median [interquartile range] and categorical variables as absolute numbers (percentages). BMI: body mass index; COPD: chronic obstructive pulmonary disease; ACE-Inhibitors: Angiotensin-Converting Enzyme Inhibitors; NSAIDs: non-steroidal anti-inflammatory drugs.

Characteristics	Without previous SARS-CoV-2 infection (LIFE Cov-, *n* = 47)	With previous SARS-CoV-2 infection (Cov+, *n* = 215)		*P*-value	LIFE Cov- vs. LIFE Cov+	LIFE Cov- vs. UOC PCS	UOC PCS vs. LIFE Cov+
		LIFE Cov+ (*n* = 115)	UOC PCS (*n* = 100)				
Age, years	50 [42; 60]	58 [53; 67]	45 [35; 53]	< 0.001	< 0.001	0.034	< 0.001
BMI, kg/m²	25.4 [23.4; 30.1]	26.8 [23.5; 29.9]	27.5 [23.4; 31.3]	0.284			
Female, n (%) of the study population	26 (55.3)	67 (58.3)	65 (65.0)	0.424			
Last infection, weeks	-	38.8 [31.2; 44.8]	36.1 [29.0; 41.4]	0.068			
Comorbidities, n (%)							
Hypertension	10 (21.3)	57 (49.6)	32 (32)	0.001	< 0.001	0.240	0.012
Myocardial infarction	1 (2.1)	2 (1.7)	2 (2.0)	1.000			
Stroke	0 (0.0)	2 (1.7)	0 (0.0)	0.664			
Heart arrhythmia	1 (2.1)	8 (7.0)	8 (8.0)	0.435			
Dyslipidemia	10 (21.3)	41 (35.7)	24 (24.0)	0.088			
Smoking	12 (25.5)	9 (7.8)	9 (9.0)	0.008	0.004	0.011	0.808
COPD	0 (0.0)	3 (2.6)	1 (1.0)	0.532			
Pneumonia	7 (14.9)	28 (24.3)	22 (22.0)	0.416			
Depression	4 (8.5)	18 (15.7)	25 (25.0)	0.039	0.314	0.025	0.091
Thyroid disease	13 (27.7)	39 (33.9)	32 (32)	0.763			
Diabetes mellitus	6 (12.8)	16 (13.9)	5 (5.0)	0.065			
Renal insufficiency	0 (0.0)	2 (1.7)	4 (4.0)	0.350			
Blood pressure, mmHg							
Systolic	123 [115; 135]	125 [118; 141]	124 [112; 133]	0.034	0.197	0.952	0.017
Diastolic	77 [71; 82]	77 [72; 83]	77 [71; 84]	0.803			
Drug intake, n (%)							
Antidepressants	3 (6.4)	9 (7.8)	10 (10.0)	0.867			
ACE-Inhibitors	4 (8.5)	9 (7.8)	10 (10.0)	0.793			
Statins	2 (4.3)	21 (18.3)	14 (14.0)	0.142			
NSAIDs	5 (10.6)	14 (12.2)	15 (15.0)	0.793			
Supplements							
Vitamins	1 (2.1)	2 (1.7)	5 (5.0)	0.426			
Omega fatty acids	1 (2.1)	2 (1.7)	1 (1.0)	0.808			

The MFI-20 was assessed in the UOC PCS, LIFE Cov+, and LIFE Cov− cohorts to evaluate SARS-CoV-2-associated fatigue (Table 2). Participants in the UOC PCS cohort reported elevated fatigue scores in the total score of the MFI-20 (median 59) compared with the LIFE Cov+ cohort (median 46) and the LIFE Cov- cohort (median 35). Additionally, this group showed higher scores across fatigue subscales, including “general fatigue”, “physical fatigue”, “reduced activity”, “mental fatigue” and “reduced motivation” subscale. These findings distinguished the UOC PCS group from both the LIFE Cov + and LIFE Cov- cohorts, whereas differences between LIFE Cov + and LIFE Cov- cohorts were limited to MFI-20 “general fatigue” and “physical fatigue” subscales. The observed differences between UOC PCS and LIFE Cov+ were expected due to the different recruitment channels. Participants were included in the LIFE Cov+ cohort if they participated in the LIFE-Adult study and had a self-reported history of SARS-CoV-2 infection. Participants in the UOC PCS cohort attended the outpatient clinic due to subjective persistent symptoms, whereas inclusion in the LIFE Cov+ cohort was based on a history of SARS-CoV-2 infection, irrespective of symptom status.

**Table 2:** Neuropsychiatric assessment of the study population consisting of participants with previous SARS-CoV-2 infections (Cov+, *n* = 215) including LIFE Cov+ (*n* = 115) and UOC PCS (*n* = 100) and controls without previous infection with SARS-CoV-2 (Cov-, *n* = 47). Differences between the groups were analysed using the Kruskal-Wallis test for continuous variables followed by Dunn’s test for post-hoc pairwise comparisons. Fisher’s exact test was used to analyze categorical variables, and pairwise Fisher tests were conducted as post hoc tests. Continuous variables are presented as median [interquartile range] and categorical variables as absolute numbers (percentages). MFI-20: Multidimensional Fatigue Inventory-20; PHQ-15: Patient Health Questionnaire-15; CESD: Center for Epidemiologic Studies Depression Scale; GAD-7: Generalized Anxiety Disorder-7.

Characteristics	Without previous SARS-CoV-2 infection (LIFE Cov-, *n* = 47)	With previous SARS-CoV-2 infection (Cov+, *n* = 215)		*P*-value	LIFE Cov- vs. LIFE Cov+	LIFE Cov- vs. UOC PCS	UOC PCS vs. LIFE Cov+
		LIFE Cov+ (*n* = 115)	UOC PCS (*n* = 100)				
Neuropsychiatric assessment							
MFI-20 Fatigue, points	35 [29; 46]	46 [34; 57]	59 [45; 75]	**< 0.001**	**0.022**	**< 0.001**	**< 0.001**
General fatigue, points	8 [6; 10]	10 [7; 13]	15 [11; 17]	**< 0.001**	**0.011**	**< 0.001**	**< 0.001**
Physical fatigue, points	7 [6; 9]	9 [6; 12]	13 [9; 17]	**< 0.001**	**0.004**	**< 0.001**	**< 0.001**
Reduced activity, points	8 [6; 10]	9 [6; 11]	13 [9; 16]	**< 0.001**	0.098	**< 0.001**	**< 0.001**
Reduced motivation, points	7 [5; 9]	8 [6; 11]	9 [6; 12]	**0.020**	0.079	**0.008**	0.370
Mental fatigue, points	7 [5; 10]	9 [6; 11]	12 [10; 16]	**< 0.001**	0.257	**< 0.001**	**< 0.001**
PHQ-15 Somatization, points	4 [2; 8]	7 [4; 10]	13 [5; 18]	**< 0.001**	**0.051**	**< 0.001**	**< 0.001**
Fatigue, n (%)	3 (6.4)	23 (20.0)	53 (53.0)	**< 0.001**	**0.034**	**< 0.001**	**< 0.001**
Headache, n (%)	3 (6.4)	5 (4.3)	25 (25.0)	**< 0.001**	0.693	**0.007**	**< 0.001**
Dizziness, n (%)	0 (0.0)	4 (3.5)	11 (11.0)	**0.008**	0.323	**0.016**	**0.033**
Sleep problems, n (%)	9 (19.1)	28 (24.3)	46 (46.0)	**< 0.001**	0.534	**0.002**	**< 0.001**
Palpitations, n (%)	1 (2.1)	3 (2.6)	12 (12.0)	**0.010**	1.000	0.061	**0.013**
Dyspnoe, n (%)	1 (2.1)	12 (10.4)	26 (26)	**< 0.001**	0.111	**< 0.001**	**0.004**
CESD Depression, points	8 [6; 12]	10 [5; 15]	15 [10; 21]	**< 0.001**	0.264	**< 0.001**	**< 0.001**
GAD-7 Anxiety, points	2 [1; 5]	3 [1; 6]	5 [3; 8]	**< 0.001**	0.481	**< 0.001**	**< 0.001**

**Fig. 2:**
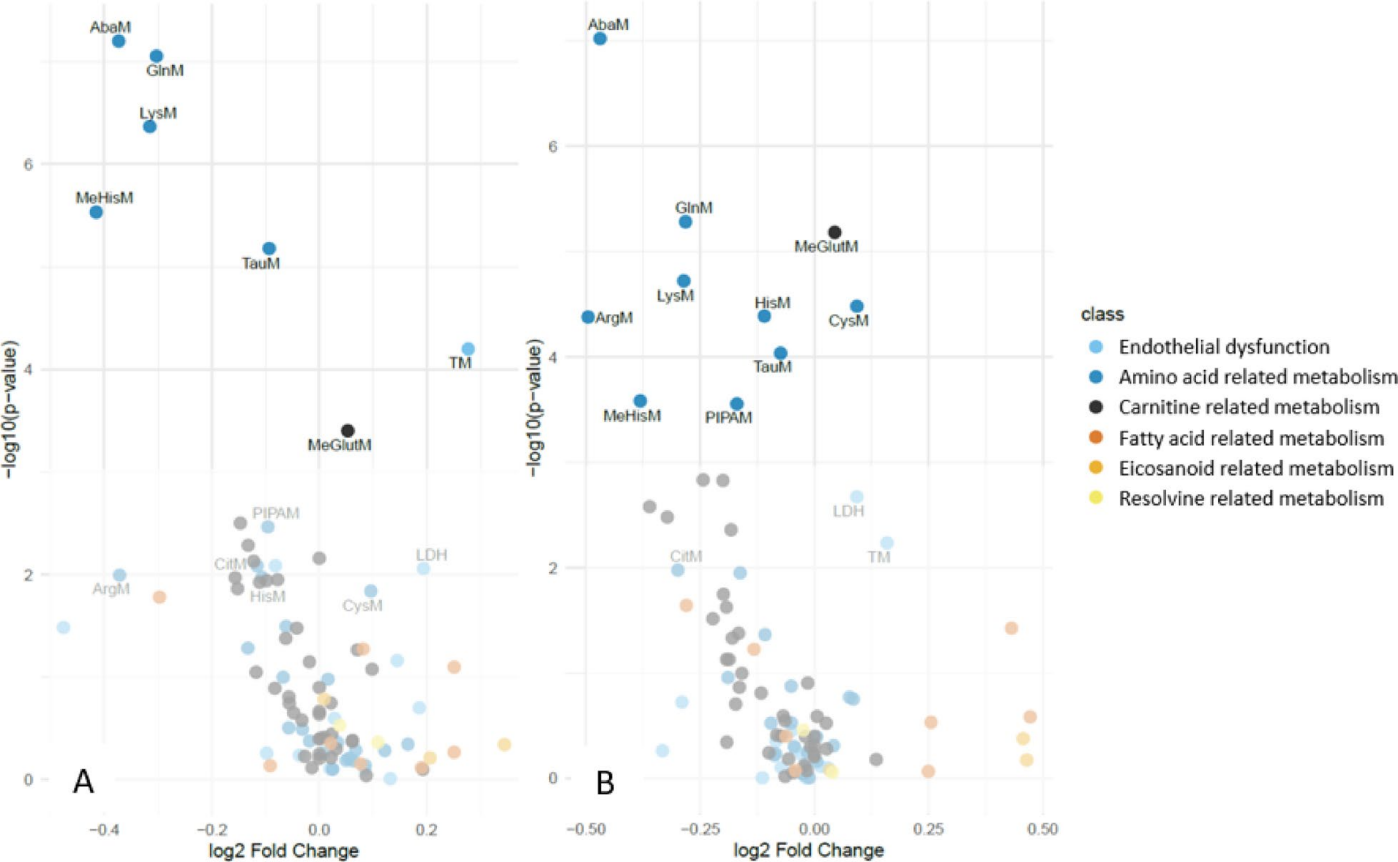
Visualization of altered biomarkers associated with previous SARS-CoV-2 infection. (**A**) Volcano plot visualizes the log2 fold changes (x-axis) against the -log10 of p-values (y-axis) of altered biomarkers in LIFE Cov+; (**B**) Volcano plot visualizes the log2 fold changes against the -log10 of p-values of altered biomarkers in UOC PCS. Color-coded metabolites were analysed using a linear model, adjusted for age, current smoking and hypertension. The false discovery rate was controlled with the Benjamini-Hochberg correction. Abbreviations: AbaM: Aminobutyric acid; GlnM: Glutamine; LysM: Lysine; TauM: Taurine; ArgM: L-Arginine; MeHisM: Methyl-Histidine; HisM: Histidine; CysM: L-Cysteine; PIPAM: Pipecolic acid; 3-MeGlutM: 3-Methylglutarylcarnitine; LDH: L-Lactatdehydrogenase; TM: soluble Thrombomodulin; CitM: Citrulline; LIFE Cov+: Leipzig Research Center for Civilization Diseases; UOC PCS: university outpatient clinic with post-COVID syndrome.

**Fig. 3:**
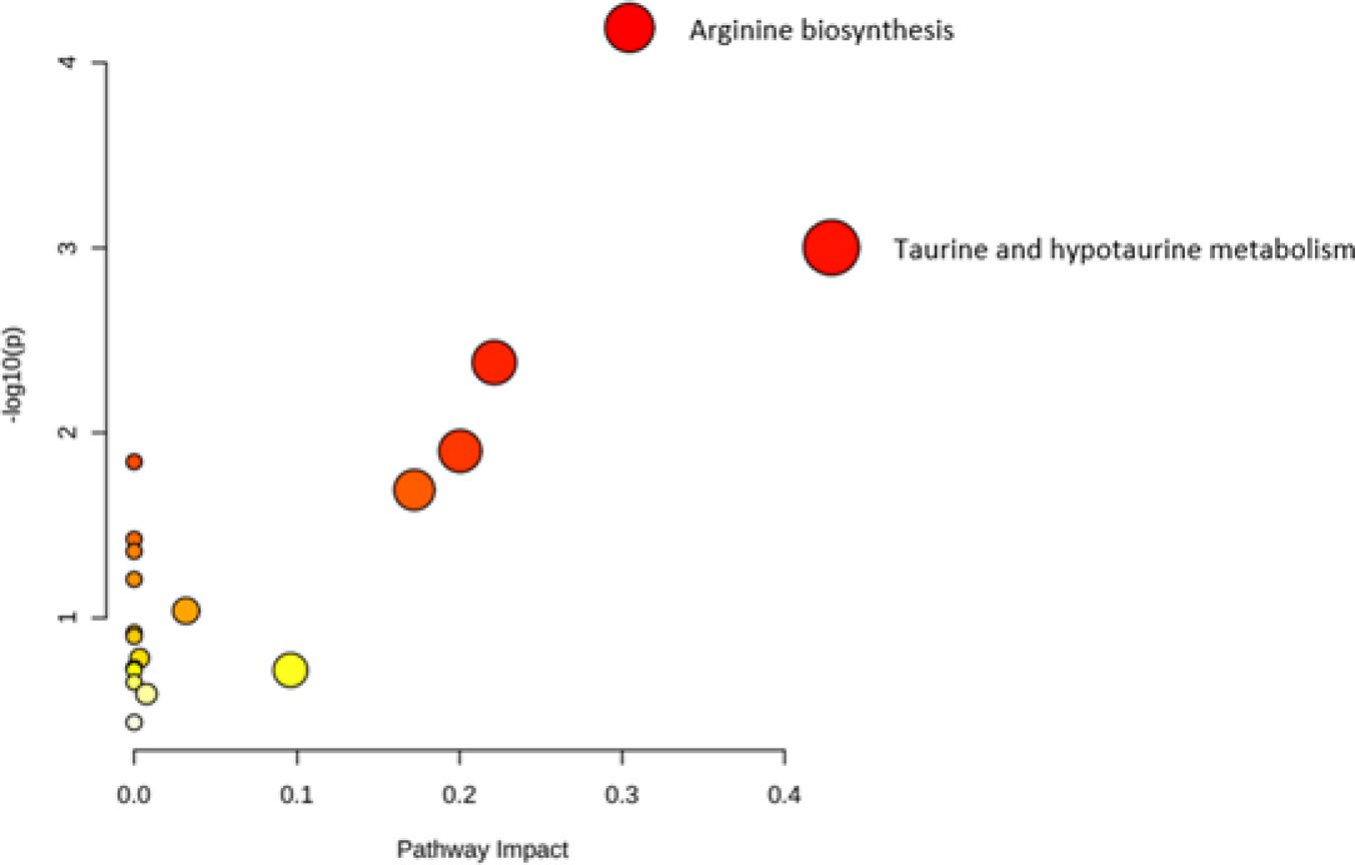
Previous SARS-CoV-2 infection is associated with decreased arginine biosynthesis and taurine and hypotaurine metabolism. Metabolic pathway analysis demonstrates pathways that were most strongly altered in subjects with previous SARS-CoV-2 infection at a median of 37.4 weeks after infection. Dots present identified pathways. The pathway impact is presented by size and p-value by color. Only significant results after Benjamini-Hochberg correction are labeled.

We compared biomarkers related to ED and amino acid, fatty acid, carnitine, eicosanoid and resolvin related metabolism between UOC PCS, LIFE Cov + and LIFE Cov-, while accounting for the confounding factors age, smoking status and hypertension. Participants with previous SARS-CoV-2 infection (UOC PCS and LIFE Cov+) exhibited higher soluble thrombomodulin (TM) and L-lactate dehydrogenase (LDH) levels compared with the LIFE Cov− cohort. Additionally, lower levels of aminobutyric acid, L-arginine, glutamine, histidine, lysine, methyl-histidine, taurine, pipecolic acid, and citrulline, along with higher L-cysteine concentrations, were observed. In carnitine metabolism, 3-methylglutarylcarnitine levels were higher in UOC PCS and LIFE Cov+ cohorts (Table 3, Supplementary Figure S1). The analysed amino acids and acylcarnitines, along with their respective metabolic pathways, are presented in Supplementary Table 3. The analysis did not reveal significant differences between the UOC PCS and LIFE Cov+ cohorts, except for L-cysteine and small dense low-density lipoprotein (sdLDL) (Table 3, Supplementary Figure S1). L-arginine and aminobutyric acid showed the largest negative fold changes among amino acids, while the endothelial marker TM and the tissue damage marker LDH displayed the largest positive fold changes (Fig. 2). No significant sex-specific differences were observed for the identified biomarkers in either the LIFE Cov + or the UOC PCS group. Correlation analysis revealed moderate or strong correlations within the identified amino acids, suggesting co-regulation within the metabolic pathways (Supplementary Figure S3). We performed a pathway analysis to further investigate the amino acid changes and identified alterations in the arginine biosynthesis and taurine, hypotaurine metabolism (Fig. 3, Supplementary Figure S2).

**Table 3:** Differences of identified biomarkers between study population consisting of participants with previous SARS-CoV-2 infections (Cov+, *n* = 215) including LIFE Cov+ (*n* = 115) and UOC PCS (*n* = 100) and controls without previous infection with SARS-CoV-2 (Cov-, *n* = 47). Differences were analysed using linear regression, adjusted for age, current smoking status and hypertension. The false discovery rate (FDR) was controlled using the Benjamini-Hochberg correction. Data are presented as median [interquartile range].

	Without previous SARS-CoV-2 infection (LIFE Cov-, *n* = 47)	With previous SARS-CoV-2 infection (Cov+, *n* = 215)		*P*-Value	*P*-Value adjusted	*P*-Value for pairwise testing		
		LIFE Cov+ (*n* = 115) UOC PCS (*n* = 100)				LIFE Cov- vs. LIFE Cov+	LIFE Cov- vs. UOC PCS	UOC PCS vs. LIFE Cov+
Endothelial dysfunction								
Thrombomodulin, pg/ml	3453 [3261; 3943]	4185 [3678; 4786]	3855 [3446; 4350]	**< 0.001**	**0.004**	**< 0.001**	**0.006**	0.141
L-Lactatdehydrogenase, µkat/l	2.71 [2.47; 3.03]	3.10 [2.83; 3.41]	2.89 [2.64; 3.36]	**0.006**	**0.040**	**0.009**	**0.002**	0.572
Small Dense Low-Density Lipoprotein, mmol/l	0.892 [0.601; 1.146]	1.015 [0.776; 1.361]	0.730 [0.583; 1.037]	**0.011**	**0.046**	0.199	0.189	**0.003**
Amino acid related metabolism								
Aminobutyric acid, µmol/l	14.1 [12.5; 16.4]	10.9 [8.7; 13.2]	10.2 [8.4; 12.5]	**< 0.001**	**< 0.001**	**< 0.001**	**< 0.001**	0.966
L-Arginine, µmol/l	36.7 [30.7; 42.0]	28.4 [22.3; 35.9]	26.0 [19.7; 34.6]	**< 0.001**	**0.001**	**0.010**	**< 0.001**	0.061
Glutamine, µmol/l	107.8 [90.6; 125.0]	87.4 [76.1; 99.9]	88.6 [77.1; 101.8]	**< 0.001**	**< 0.001**	**< 0.001**	**< 0.001**	0.342
L-Cysteine, µmol/l	855 [821; 902]	913 [850; 999]	911 [869; 969]	**< 0.001**	**< 0.001**	**0.015**	**< 0.001**	**0.037**
Histidine, µmol/l	51.3 [46.9; 56.2]	47.7 [43.7; 52.7]	47.6 [41.6; 52.6]	**< 0.001**	**< 0.001**	**0.011**	**< 0.001**	0.058
Lysine, µmol/l	56.1 [48.5; 70.0]	45.1 [39.0; 52.0]	46.0 [39.6; 55.7]	**< 0.001**	**< 0.001**	**< 0.001**	**< 0.001**	0.354
Methyl-Histidine, µmol/l	0.056 [0.052; 0.064]	0.042 [0.034; 0.052]	0.043 [0.037; 0.057]	**< 0.001**	**< 0.001**	**< 0.001**	**< 0.001**	0.222
Taurine, µmol/l	8.3 [8.0; 8.7]	7.8 [7.5; 8.2]	7.9 [7.4; 8.3]	**< 0.001**	**< 0.001**	**< 0.001**	**< 0.001**	0.488
Pipecolic acid, µmol/l	6.5 [5.4; 8.1]	6.1 [5.3; 7.2]	5.8 [4.7; 6.5]	**< 0.001**	**0.003**	**0.003**	**< 0.001**	0.376
Citrulline, µmol/l	27.9 [23.5; 30.7]	25.7 [21.6; 28.4]	22.6 [19.7; 26.5]	**0.016**	**0.046**	**0.008**	**0.011**	0.938
Carnitine related metabolism								
3-Methylglutarylcarnitine, µmol/l	0.080 [0.077; 0.082]	0.083 [0.080; 0.085]	0.082 [0.079; 0.086]	**< 0.001**	**< 0.001**	**< 0.001**	**< 0.001**	0.222

**Fig. 4:**
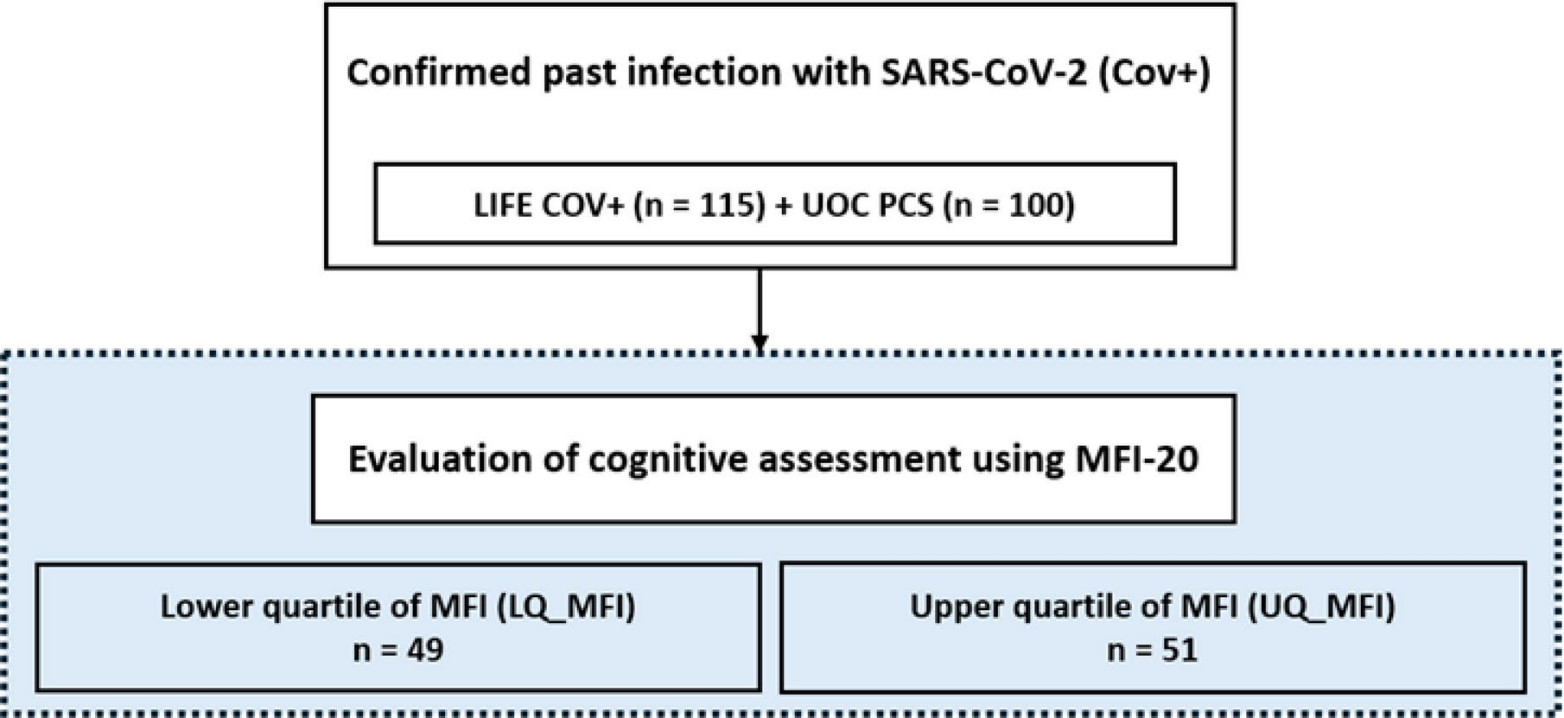
Study scheme to investigate the association of fatigue severity in participants with previous infection with SARS-CoV-2 with biomarkers for ED and amino acid, fatty acid, carnitine, eicosanoid and resolvin related metabolism. A total of 115 study participants from the LIFE study (LIFE Cov+) and 100 patients from the university outpatient clinic (UOC PCS), who met the inclusion and exclusion criteria for the group with previous infection with SARS-CoV-2, were further assessed for fatigue severity using the Multidimensional Fatigue Inventory (MFI-20). The upper and the lower quartiles comprising 51 and 49 individuals with the highest (UQ_MFI) or lowest fatigue scores (LQ_MFI) were analysed to identify differences associated with fatigue-related post-COVID symptoms.

We investigated the association between fatigue-related PCS symptom load and measured biomarkers of ED and amino acid, fatty acid, carnitine, eicosanoid and resolvin related metabolism. Fatigue severity was assessed using the MFI-20. Both groups, UOC PCS and LIFE Cov+, were combined for this analysis (Fig. 4). Based on their MFI-20 scores, participants were stratified into upper (UQ_MFI, *N* = 51) and lower (LQ_MFI, *N* = 49) quartiles of fatigue severity to identify PCS-associated biomarkers after SARS-CoV-2 infection (Fig. 4).

Participants in the UQ_MFI group were slightly younger (median 51 years) than those in the LQ_MFI group (median 57 years). Additionally, the UQ_MFI cohort exhibited a higher body mass index (BMI, 28.8 kg/m²) compared with the LQ_MFI cohort (26.6 kg/m²). As expected, depression was more prevalent in the UQ_MFI cohort (43.1%) compared with the LQ_MFI cohort (8.2%) (Supplementary Table 1a) and participants in the UQ_MFI cohort reported higher levels of fatigue across all five subscores: general fatigue, physical fatigue, reduced activity, reduced motivation, and mental fatigue (Supplementary Table 1b). Regarding examined biomarkers, we identified elevated levels of the polyunsaturated fatty acid (PUFA) linoleic acid (LA), as well as monounsaturated fatty acids (MUFAs) oleic acid (OA) and palmitoleic acid (PA), while adjusting for the confounding factors age and body mass index in participants in the UQ_MFI group (Table 4, Supplementary Fig. S4). In addition, no significant sex-specific differences were observed in the identified biomarkers within either the LQ_MFI or the UQ_MFI cohort. Correlation analysis revealed strong correlations within the identified fatty acids (Supplementary Figure S5). Since the observed differences in fatty acid metabolism could be influenced by nutritional supplementation, we additionally analysed the intake of omega fatty acids and vitamins. No significant differences were found between the groups, suggesting that intake of nutritional supplements is unlikely to explain the observed effects.

**Table 4:** Serum fatty acids levels are elevated in subjects with low or high fatigue-related PCS symptoms according to MFI assessment. Differences between the groups were analysed using linear regression, adjusted for age, body mass index and depression status. The false discovery rate was controlled using the Benjamini-Hochberg correction. Data are presented as median [interquartile range]. MFI-20: Multidimensional Fatigue Inventory-20.

	Lower quartile of MFI (*n* = 49) in median [IQR]	Upper quartile of MFI (*n* = 51) in median [IQR]	*P*-Value	*P*-Value adjusted
Linoleic acid, µg/ml	13.1 [8.1; 21.0]	22.3 [13.3; 31.7]	0.008	0.030
Oleic acid, µg/ml	17.1 [9.8; 22.4]	25.2 [17.7; 31.0]	0.004	0.030
Palmitoleic acid, µg/ml	2.6 [1.7; 4.3]	5.2 [2.6; 7.7]	0.011	0.030

## Discussion

Given the clinical heterogeneity of PCS and the lack of specific physical findings, suitable biomarkers would provide valuable assistance for establishing a diagnosis, assessing disease severity, and monitoring PCS – among these, plasma biomarkers are attractive candidates due to the non-invasive nature and practical feasibility of their collection and analysis. Since acute SARS-CoV-2 infection is associated with varying degrees of ED, inflammation, and metabolic alterations, we used our well-characterized cohort, examined at a median of 37.4 weeks post-acute infection, to explore blood biomarkers (ED, amino acid, fatty acid, carnitine, eicosanoid and resolvin metabolism). Initially, we compared individuals with previous SARS-CoV-2 infection with those without. Our findings revealed higher levels of soluble thrombomodulin (TM), L-lactate dehydrogenase (LDH), and specific amino acid alterations related to nitric oxide (NO) metabolism, as well as increased levels of 3-methylglutarylcarnitine (3-MeGluM). Interestingly, no significant differences in these parameters were observed between after SARS-CoV-2 infection. However, participants with higher PCS-related fatigue severity exhibited increased levels of the PUFA LA and the MUFAs OA and PA.

LDH has been identified as an important biomarker in the context of acute SARS-CoV-2 infection and PCS, as highlighted in the meta-analysis by Martha et al. and Yong et al.^2,35^. Our findings show that elevated LDH levels persist for months post-infection independent of PCS related symptoms as measured by the MFI-20. TM is recognized as a marker of endothelial injury and thromboinflammation and has been proposed as a biomarker for ED and thrombotic risk in severe COVID-19 cases^36^. Further research, such as that by Fogarty et al. shows persistent elevation of TM and other markers such as von Willebrand factor antigen and von Willebrand factor propeptide, has also been observed in convalescent COVID-19 patients, indicating ongoing ED^9^. Our results confirm the persistent elevation of TM post SARS-CoV-2 infection. Notably, the increase in TM is independent of PCS-related fatigue severity.

Analysis of serum levels of metabolites reflecting amino acid metabolism revealed that almost 40% of markers were altered in individuals post-acute infection, suggesting a persistent and substantial change of the amino acid metabolism. Pathway analysis demonstrated decreases in arginine and taurine and hypotaurine metabolism. L-arginine and taurine are critical in nitric oxide (NO) metabolism, which is essential for endothelial function and vascular homeostasis. NO, primarily synthesized from L-arginine by endothelial nitric oxide synthase (eNOS), regulates blood pressure and vascular homeostasis^37^. Taurine modulates NO bioavailability and mitigates oxidative stress through its antioxidant effects, which include inhibiting reactive oxygen species (ROS) production^38^. Our data indicates that SARS-CoV-2 infection leads to persistent alterations in NO metabolism at a median of 37.4 weeks post-acute infection, potentially promoting ED and vascular complications. These findings are consistent with other studies that have reported long-term metabolic alterations in post-COVID patients specifically. For instance, persistent alterations in arginine metabolism were observed 12 months post-infection in non-hospitalized post-COVID patients^10^, and prolonged alterations in taurine and hypotaurine metabolism for up to two years post-infection^6^. Lower plasma L-arginine and taurine levels have also been associated with PCS disease severity, suggesting a significant role for both in modulating PCS symptomatology^6,39^. Surprisingly, our cohort displayed no association between taurine or L-arginine and fatigue-related PCS severity. Amino acids such as citrulline, glutamine, and cysteine, involved in arginine biosynthesis and taurine and hypotaurine metabolism, have previously been linked to NO metabolism and antioxidant protection in cell culture and clinical observation^40–42^. Analysis of carnitine metabolism-related serum metabolites revealed elevated 3-MeGluM levels following infection, which have also been observed in severe COVID-19 cases, suggesting a link between increased fatty acid oxidation and other metabolic disturbances^43^. It has been shown that TM influences NO signaling by inducing Ca²⁺-dependent activation of eNOS, thereby enhancing NO production^44^. Since amino acids such as L-arginine and homocysteine are key regulators of NO biosynthesis and their altered metabolism is closely linked to ED^7,8^ we investigated the link between TM and amino acids.

However, no correlation was found between the significantly altered amino acids and either TM, LDH or 3-MeGluM, except between L-cysteine and LDH in UOC PCS suggesting that these biomarkers may reflect independent pathophysiological mechanisms. The observed metabolic changes could be due to altered nutritional intake of amino acids. As there were no differences in nutritional supplement intake, we conclude that the observed changes were due to previous SARS-CoV-2 infection rather than differences in nutritional supplement intake.

The comparison of subjects with low and high fatigue severity, as quantified by the MFI-20 scale, showed higher levels of the PUFA LA and the MUFAs OA and PA among participants with higher fatigue severity. Interestingly, SARS-CoV-2-asscociated biomarkers were not altered between subjects with high and low disease severity. Notably, 43.1% of these highly impacted participants were diagnosed with depression, pointing to a potential link between observed fatty acid elevations and mental health. Prior studies have already shown a relationship between LA and depression, indicating that higher serum levels of LA and an increased omega-6 to omega-3 PUFA ratio, may contribute to an increased risk of depression and other psychological disorders^45,46^. Accordingly, elevated LA levels have been associated with inflammation and mitochondrial dysfunction, which contribute to PCS associated symptoms such as fatigue^47^. Such metabolic changes in PCS patients were observed to persist for up to two years after COVID-19 recovery^6^, suggesting a specific role in PCS pathology^48^. In addition to LA, our study identified elevated levels of PA and OA in participants with higher fatigue severity, consistent with findings that report similar results in patients post acute SARS-COV 2 infection^47^. Guntur et al. have shown that these fatty acid elevations are caused by COVID-asscociated mitochondrial dysfunction. Mitochondrial dysfunction in long COVID likely impairs energy production, erythrocyte survival and oxygen transport, potentially contributing to symptoms such as fatigue and dyspnea^47^. Our results corroborate these insights and demonstrate that changes in fatty acid metabolism, particularly the elevated levels of the PUFA LA and the MUFAs OA and PA, are associated with PCS-related fatigue severity and persist at a median of 37.4 weeks after SARS-CoV-2 infection.

While our study benefits from having the largest cohort size to date for the biomarkers measured, several limitations should be acknowledged. Laboratory parameters were assessed only once, without any baseline measurements taken during the acute phase of the infection, limiting our ability to track changes over time from the onset of infection. Infection data were based on patient-reported history, which may introduce recall bias. This study focused primarily on the fatigue dimension of PCS, without systematic assessment of other symptom domains such as post-exertional malaise, brain fog, autonomic dysfunction, or sleep disturbances. Therefore, the findings reflect fatigue-specific aspects and may not be generalizable to the full spectrum of post-COVID symptoms. Another limitation is the lack of access to dietary data. However, there were no differences between the groups regarding the frequency of supplement intake.

Furthermore, all participants were recruited from the Leipzig metropolitan area, potentially limiting the generalizability of our findings, as it does not account for potential ethnic and genetic variations that could influence biomarker levels and PCS symptoms. Adjustment for depressive symptoms was not undertaken due to substantial conceptual overlap between depression-related symptoms and the dimensions assessed by the MFI-20. Future studies should adopt a longitudinal design, with long-term follow-up measurements that will provide a more comprehensive understanding of the observed changes over time.

In summary, our findings show that changes in biomarkers indicative of ED, including alterations in arginine and taurine-related nitric oxide (NO) metabolism, persist at a median of 37.4 weeks following SARS-CoV-2 infection. Additionally, our findings revealed that fatigue-related PCS severity is associated with persistent specific fatty acid elevations, suggesting their potential utility as biomarkers for establishing a diagnosis, assessing disease severity, and monitoring PCS.

## Supplementary Information

Below is the link to the electronic supplementary material.

Supplementary Material 1

## Data Availability

The datasets used and/or analysed during the current study available from the corresponding author on reasonable request.
