# Supplementary material for: Endothelial dysfunction and metabolic biomarkers in post-COVID-19 syndrome
Source: Sci Rep. 2026 May 13;16:15067. doi: 10.1038/s41598-026-50965-6 (PMC13172428; doi:10.1038/s41598-026-50965-6)
Supplement: Supplementary file 1 — Supplementary Material 1 [file 41598_2026_50965_MOESM1_ESM.docx]

**Supplementary Materials**

| 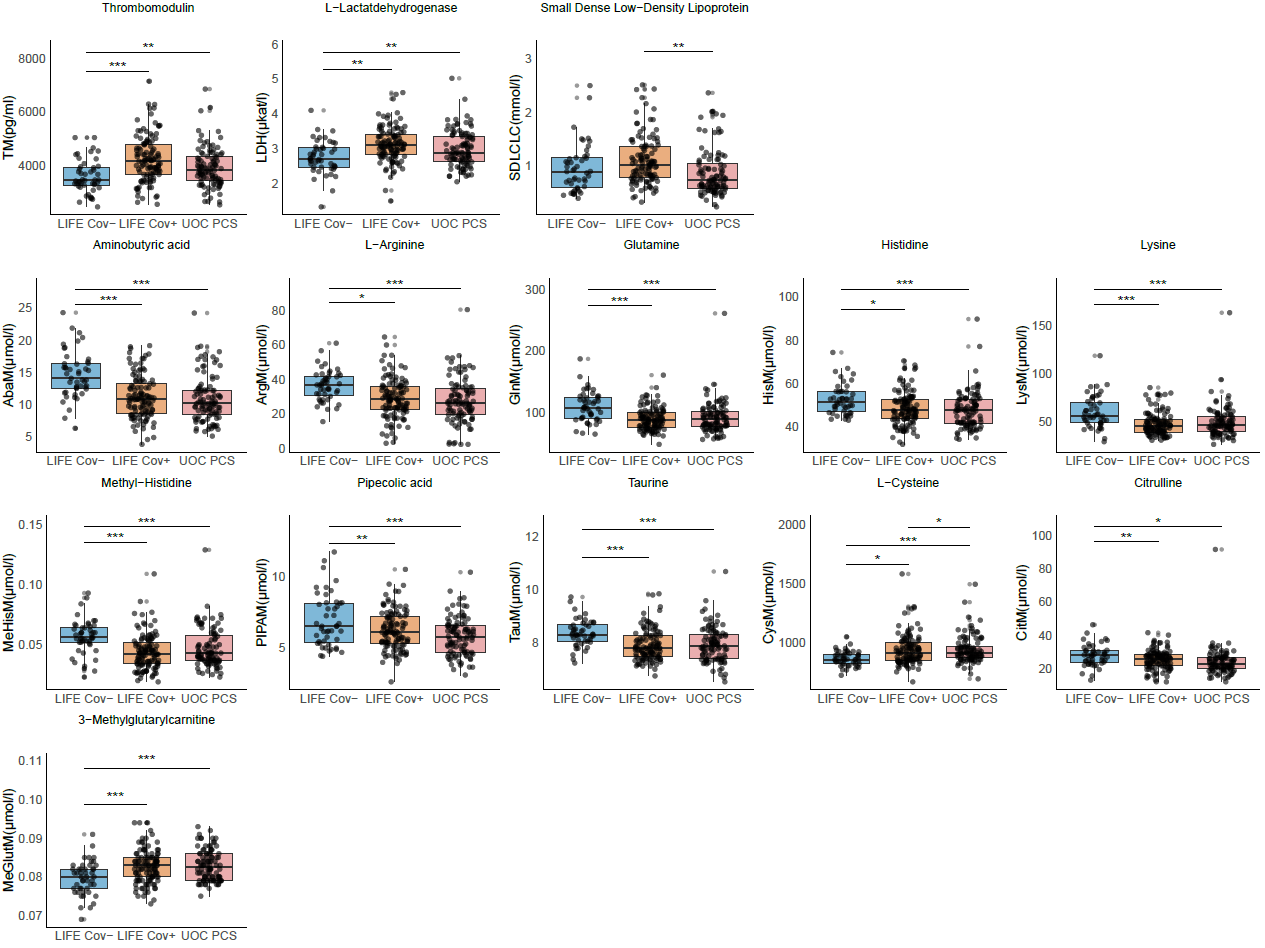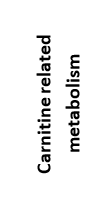**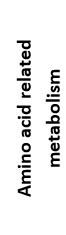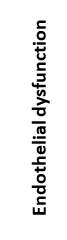** |
| --- |
| \| **Supplementary Figure S1: Alterations in biomarkers for ED, amino acid and carnitine related metabolism in subjects with previous SARS-CoV-2 infection**. Markers of ED and serum levels of amino acid and carnitine related metabolism are altered in individuals with previous SARS-CoV-2 infection. Comparison between the control group with no previous infection with SARS-CoV-2 (n=47, labeled as "LIFE Cov-“), the LIFE Cov+ group (n= 115) and the UOC PCS group (n= 100). Boxplots display the data as originally measured. Statistical significance indicated by asterisks: *p < 0.05, **p < 0.01**,** ***p < 0.001 was analysed using linear regression, adjusted for confounding variables including age, current smoking and hypertension. The false discovery rate was controlled with the Benjamini-Hochberg correction. LIFE Cov-: Leipzig Research Center for Civilization Diseases Covid negative; LIFE Cov+: Leipzig Research Center for Civilization Diseases Covid positive; UOC PCS: university outpatient clinic with post-COVID syndrome \| \| --- \|   **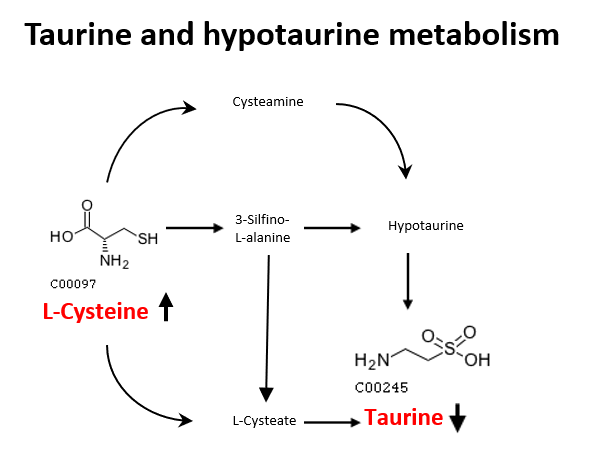**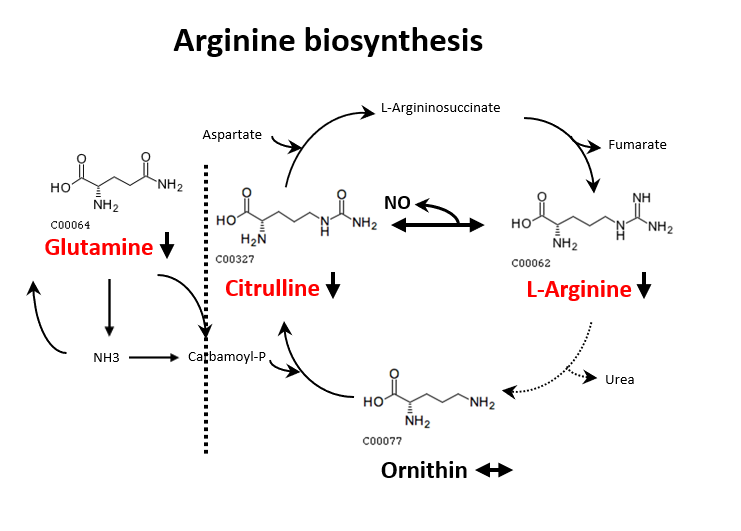 |
| **Supplementary Figure S2: Altered amino acids in previous SARS-CoV-2 infection associated metabolic pathways.** Serum levels of L-arginine, citrulline and glutamine in arginine biosynthesis and serum levels of L-cysteine and taurine in taurine and hypotaurine metabolism are altered in individuals with previous SARS-CoV-2 infection. KEGG pathway analysis of metabolic pathways was conducted using MetaboAnalyst 6.0, focusing on the integration of metabolomic data with annotated biochemical pathways. |

| **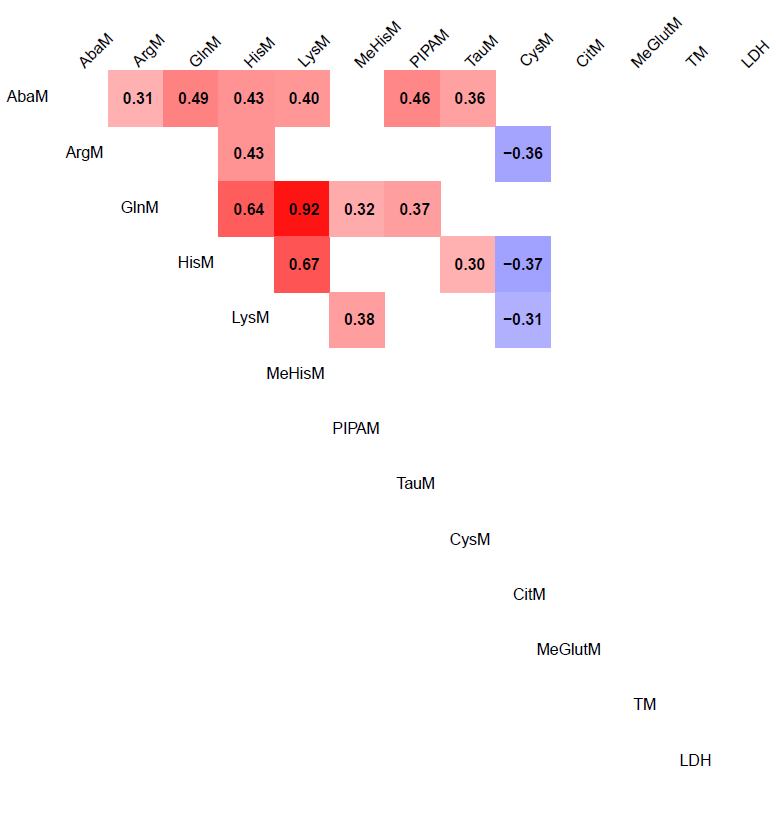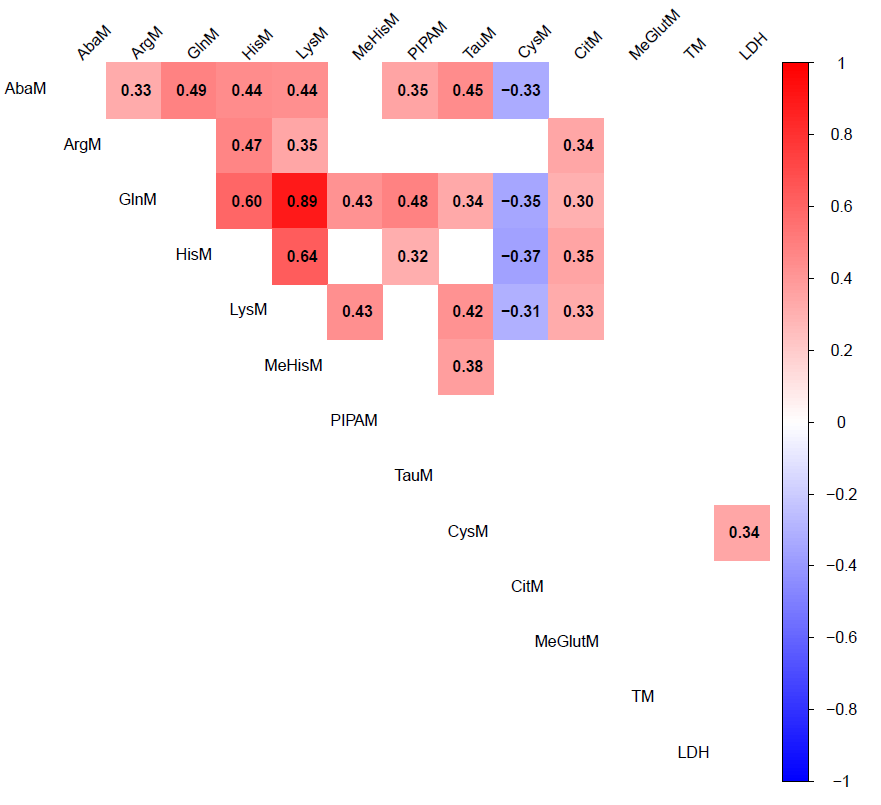**  B  A |
| --- |
| **Supplementary Figure S3: Correlation analysis of identified biomarkers associated with previous SARS-CoV-2 infection.** A) The correlation shows pairwise Spearman correlations between identified biomarkers in LIFE Cov+; B) The correlation shows pairwise Spearman correlations between identified biomarkers in UOC PCS. Only correlations with absolute values greater than >\|0.3\|and p < 0.05 are shown. Color scale represents the strength of the correlation of the identified biomarkers. AbaM: Aminobutyric acid; ArgM: L-Arginine; GlnM: Glutamine; HisM: Histidine; LysM: Lysine; MeHisM: Methyl-Histidine; PIPAM: Pipecolic acid; TauM: Taurine; CysM: L-Cysteine; CitM: Citrulline; MeGlutM: Methylglutarylcarnitine; TM: soluble Thrombomodulin; LDH: L-Lactatdehydrogenase; LIFE Cov+: Leipzig Research Center for Civilization Diseases; UOC PCS: university outpatient clinic with post-COVID syndrome |

| **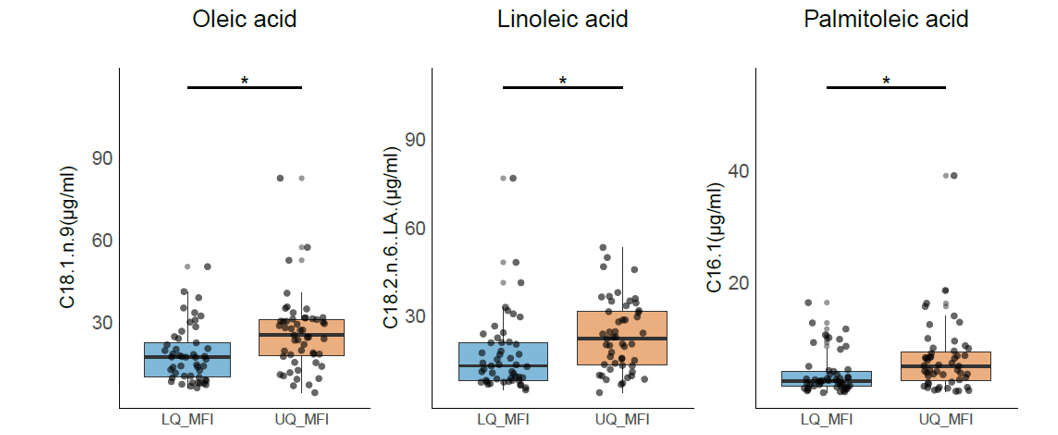** |
| --- |
| **Supplementary Figure S4:** **Serum fatty acids are elevated in participants with high** **fatigue-related post-COVID symptoms.** Oleic acid, Linoleic acid and Palmitoleic acid are increased in individuals with previous SARS-CoV-2 infection with high fatigue-related post-COVID symptoms. Comparison between subjects with low fatigue-related post-COVID symptoms (n=49, labeled as “LQ_MFI“, orange box plot) and high fatigue-related post-COVID symptoms (n=51, labeled as “UQ_MFI“, blue box plot) according to MFI assessment. Boxplots display the data as originally measured. Statistical significance indicated by asterisks: *p < 0.05, **p < 0.01**,** ***p < 0.001 was analysed using a linear model, adjusted for confounding variables age, body mass index and depression status. The false discovery rate was controlled with the Benjamini-Hochberg correction. MFI: Multidemsional Fatigue Inventory   \| 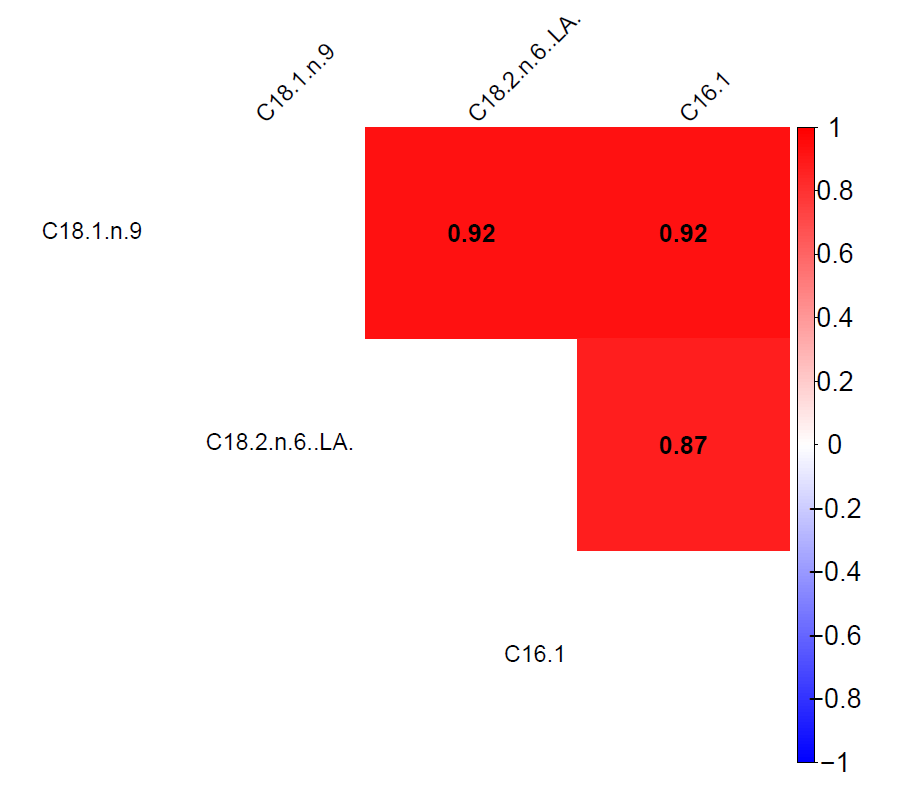 \| \| --- \| \| **Supplementary Figure S5: Correlation analysis of identified biomarkers associated with high fatigue-related post-COVID symptoms.** The correlation shows pairwise Spearman correlations between identified biomarkers. Only correlations with absolute values greater than >\|0.3\| and p < 0.05 are shown. Color scale represents the strength of the correlation of the identified biomarkers. C18.1.n.9: Oleic acid; C18.2.n.6..LA.: Linoleic acid; C16.1: Palmitoleic acid \|  \| **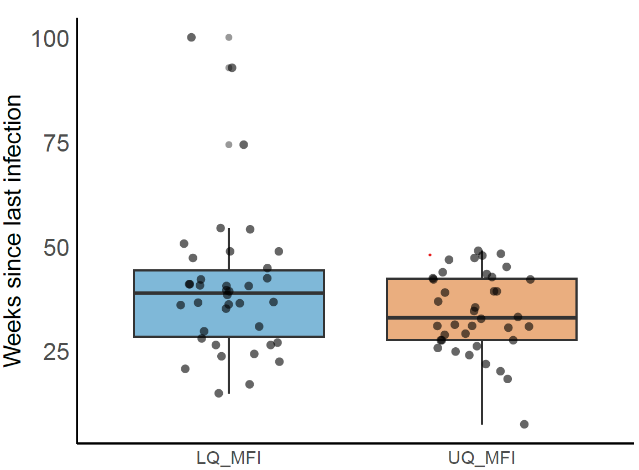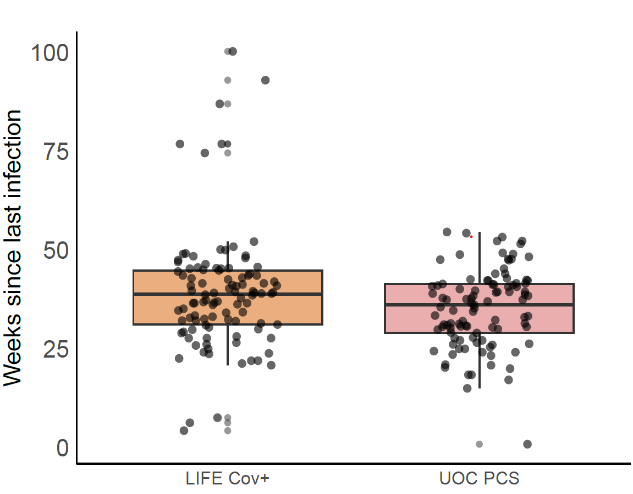** \| \| --- \| \| **Supplementary Figure S6: Similar time span since last SARS-CoV-2 infection between individuals with or without PCS and between individuals with low or high fatigue-related PCS symptoms**. Median time since infection was 38.8 weeks [31.2; 44.8] for LIFE Cov+ and 36.1 weeks [29.0; 41.4] for UOC, and 39.0 weeks [28.6; 44.4] for LQ_MFI and 33.1 weeks [27.7; 42.5] for UQ_MFI. LIFE Cov+: Leipzig Research Center for Civilization Diseases; UOC PCS: University outpatient clinic with post-COVID syndrome; LQ_MFI: lower quartile of fatigue-related post-COVID symptoms; UQ_MFI: upper quartile of fatigue-related post-COVID symptoms \|   **Supplementary Table 1a: Baseline characteristics of subjects with low or high fatigue-related post-COVID symptoms in participants with confirmed previous SARS-CoV-2 infection.** Lower (LQ_MFI, n=49) and upper quartile (UQ_MFI, n=51) of MFI assessment were compared. Differences between the groups were analysed using the Wilcoxon signed-rank test for continuous variables and the Fisher-Exact test for categorical variables. The results are presented as the median [interquartile range] for continuous variables and as absolute numbers (percentages) for categorical variables. MFI-20: Multidimensional Fatigue Inventory-20; BMI: body mass index; COPD: chronic obstructive pulmonary disease; ACE-Inhibitors: Angiotensin-Converting Enzyme Inhibitors; NSAIDs: non-steroidal anti-inflammatory drugs   \| **Characteristics** \| **Lower quartile of MFI (n=49)** \| **Upper quartile of MFI (n=51)** \| **P-value** \| \| --- \| --- \| --- \| --- \| \| Age, years \| 57 [49; 66] \| 51 [41; 57] \| **0.009** \| \| BMI, kg/m² \| 26.6 [22.5; 29.9] \| 28.8 [24.2; 33.4] \| **0.022** \| \| Female, n (%) of the study population \| 30 (61.2) \| 35 (68.6) \| 0.530 \| \| Last infection, weeks \| 39.0 [28.6; 44.4] \| 33.1 [27.7; 42.5] \| 0.224 \| \| **Comorbidities, n (%)** \|  \|  \|  \| \| Hypertension \| 21 (42.9) \| 25 (49) \| 0.554 \| \| Myocardial infarction \| 1 (2.0) \| 1 (2.0) \| 1.000 \| \| Stroke \| 0 (0.0) \| 0 (0.0) \| 1.000 \| \| Heart arrhythmia \| 0 (0.0) \| 2 (3.9) \| 0.495 \| \| Dyslipidaemia \| 12 (24.5) \| 18 (35.3) \| 0.279 \| \| Smoking \| 3 (6.1) \| 4 (7.8) \| 1.000 \| \| COPD \| 2 (4.1) \| 1 (2.0) \| 0.617 \| \| Pneumonia \| 13 (26.5) \| 14 (27.5) \| 1.000 \| \| Depression \| 4 (8.2) \| 22 (43.1) \| **<0.001** \| \| Thyroid disease \| 17 (34.7) \| 21 (41.2) \| 0.542 \| \| Diabetes mellitus \| 7 (14.3) \| 5 (9.8) \| 0.550 \| \| Renal insufficiency \| 1 (2.0) \| 2 (3.9) \| 1.000 \| \| **Blood pressure, mmHg** \|  \|  \|  \| \| Systolic \| 127 [116; 148] \| 124 [114; 134] \| 0.119 \| \| Diastolic \| 78 [73; 85] \| 79 [72; 87] \| 0.495 \| \| **Drug intake,** **n (%)** \|  \|  \|  \| \| Antidepressants \| 0 (0.0) \| 9 (17.6) \| **0.004** \| \| ACE-Inhibitors \| 3 (6.1) \| 7 (13.7) \| 0.502 \| \| Statins \| 6 (12.2) \| 13 (25.5) \| 0.298 \| \| NSAIDs \| 6 (12.2) \| 9 (17.6) \| 1.000 \| \| Supplements \|  \|  \|  \| \| Vitamins \| 1 (2.0) \| 3 (5.9) \| 0.629 \| \| Omega fatty acids \| 1 (2.0) \| 1 (2.0) \| 1.000 \| \|  \|  \|  \|  \|  \| **Supplementary Table 1b: Neuropsychiatric assessment of subjects with low or high fatigue-related post-COVID symptoms according to MFI assessment.** Lower (LQ_MFI, n=49) and upper quartile (UQ_MFI, n=51) of MFI were compared. Differences between the groups were analysed using the Wilcoxon signed-rank test for continuous variables and the Fisher-Exact test for categorical variables. The results are presented as the median [interquartile range] for continuous variables and as absolute numbers (percentages) for categorical variables. MFI-20: Multidimensional Fatigue Inventory-20; PHQ-15: Patient Health Questionnaire-15; CESD: Center for Epidemiologic Studies Depression Scale; GAD-7: Generalized Anxiety Disorder-7 \| \| \| \| \| --- \| --- \| --- \| --- \| \| **Characteristics** \| **Lower quartile of MFI (n=49)** \| **Upper quartile of MFI (n=51)** \| **P-value** \| \| **Neuropsychiatric assessment** \|  \|  \|  \| \| **MFI-20 Fatigue, points** \| 30 [26; 35] \| 77 [72; 87] \| **<0.001** \| \| General fatigue, points \| 7 [4; 8] \| 17 [16; 19] \| **<0.001** \| \| Physical fatigue, points \| 6 [5; 7] \| 17 [15; 19] \| **<0.001** \| \| Reduced activity, points \| 5 [5; 6] \| 16 [14; 18] \| **<0.001** \| \| Reduced motivation, points \| 5 [4; 6] \| 12 [10; 14] \| **<0.001** \| \| Mental fatigue, points \| 6 [4; 7] \| 16 [14; 18] \| **<0.001** \| \| **PHQ-15 Somatization, points** \| 4 [2; 7] \| 18 [13; 21] \| **<0.001** \| \| Fatigue, n (%) \| 1 (2.0) \| 47 (92.2) \| **<0.001** \| \| Headache, n (%) \| 2 (4.1) \| 18 (35.3) \| **<0.001** \| \| Dizziness, n (%) \| 1 (2.0) \| 12 (23.5) \| **0.002** \| \| Sleep problems, n (%) \| 3 (6.1) \| 35 (68.6) \| **<0.001** \| \| Palpitations, n (%) \| 0 (0.0) \| 10 (19.6) \| **0.001** \| \| Dyspnoe, n (%) \| 0 (0.0) \| 25 (49.0) \| **<0.001** \| \| **CESD Depression, points** \| 6 [4; 8] \| 23 [17; 30] \| **<0.001** \| \| **GAD-7 Anxiety, points** \| 1 [0; 4] \| 7 [5; 11] \| **<0.001** \| |

**Supplementary Table 2:**

| **Abbreviation** | **Full Name** | **Pathway** | **Superpathway** | **Category** |
| --- | --- | --- | --- | --- |
| WBC | Leukocytes | Immune Response | Inflammatory Response | endothelial dysfunction |
| CRPHS | C-Reactive Protein (High Sensitivity) | Inflammation Marker | Acute-phase Response | endothelial dysfunction |
| LDH | Lactatdehydrogenase | Glycolysis | Metabolism | endothelial dysfunction |
| TM | Soluble Thrombomodulin | Coagulation | Hemostasis | endothelial dysfunction |
| VWF | Von Willebrand factor antigen | Coagulation | Hemostasis | endothelial dysfunction |
| ICAM1 | Soluble Intercellular Adhesion Molecule 1 | Cell Adhesion | Inflammatory Response | endothelial dysfunction |
| VCAM | Soluble Vascular Cell Adhesion Molecule-1 | Cell Adhesion | Inflammatory Response | endothelial dysfunction |
| APTT | Activated partial thromboplastin time | Coagulation Cascade | Hemostasis | endothelial dysfunction |
| PT | Prothrombin time | Coagulation Cascade | Hemostasis | endothelial dysfunction |
| DDIM | D-dimers | Fibrinolysis | Hemostasis | endothelial dysfunction |
| PCA | Protein C antigen | Anticoagulation | Hemostasis | endothelial dysfunction |
| FIB | Fibrinogen | Coagulation Cascade | Hemostasis | endothelial dysfunction |
| SDLDL | Small Dense Low-Density Lipoprotein | Lipid Profile | Lipid Transport | endothelial dysfunction |
| HDL | High-Density Lipoprotein | Lipid Profile | Lipid Transport | endothelial dysfunction |
| GLU | Glucose | Energy Metabolism | Glycemic Control | amino acid related metabolism |
| ALAT | Alanine Aminotransferase | Liver Enzyme | Amino Acid Metabolism | liver metabolism |
| ASAT | Aspartate Aminotransferase | Liver Enzyme | Amino Acid Metabolism | liver metabolism |
| Aba | Aminobutyric acid | GABA, AABA | Other | amino acid related metabolism |
| Ala | Alanine | Muscle metabolism, Ammonia-carrier, Glucose metabolism | Urea cycle | amino acid related metabolism |
| Arg | Arginine | Urea cycle, NO metabolism | Urea cycle | amino acid related metabolism |
| Asn | Asparagine | Amino Acid Biosynthesis | Amino acid metabolism, other | amino acid related metabolism |
| Asp | Aspartic acid | Amino group recycling | Ammonia recycling | amino acid related metabolism |
| Carn | Carnosin | Histidine metabolism, Beta-alanine metabolism | Amino acid metabolism, other | amino acid related metabolism |
| Cit | Citrulline | Urea cycle, NO metabolism | Urea cycle | amino acid related metabolism |
| Gln | Glutamine | Amino group recycling | Ammonia recycling | amino acid related metabolism |
| Glu | Glutamic acid | Amino group recycling, GABA production | Ammonia recycling | amino acid related metabolism |
| Gly | Glycine | Serin katabolism, brain function, purine synthesis, porphyrin synthesis | Ammonia recycling | amino acid related metabolism |
| His | Histidine | Histamine precorsor, Carnosin precursor | Amino acid metabolism, other | amino acid related metabolism |
| Leu\|Ile | Leucine\|Isoleucine | Leucine metabolsim, Isoleucine metabolism | BCAA metabolism | amino acid related metabolism |
| Lys | Lysine | Carnitine precursor, collagen, glutamate generation | Amino acid metabolism, other | amino acid related metabolism |
| MeHis | Methyl-histidine | Histidine metabolism | Amino acid metabolism, other | amino acid related metabolism |
| Met | Methionine | Cytosolic methyl group transfer, intermediate in the biosynthesis of cysteine, carnitine, taurine, lecithin, phosphatidylcholine, and other phospholipids | Amino acid metabolism, other | amino acid related metabolism |
| Orn | Ornithine | Urea cycle | Urea cycle | amino acid related metabolism |
| Phe | Phenylalanine | Dopamine precursor | Amino acid metabolism, other | amino acid related metabolism |
| PiPA | Pipecolic acid | Peroxisomal metabolism, Lysine degradation | Amino acid metabolism, other | amino acid related metabolism |
| Pro | Proline | Collagen synthesis | Collagen synthesis | amino acid related metabolism |
| Sarc | Sarcosine | Glycine katabolism | Amino acid metabolism, other | amino acid related metabolism |
| Ser | Serine | Glycine formation | Ammonia recycling | amino acid related metabolism |
| Tau | Taurine | Cystein metabolism, bile acid generation | Amino acid metabolism, other | amino acid related metabolism |
| Trp | Tryptophan | Serotonine precursor | Amino acid metabolism, other | amino acid related metabolism |
| Tyr | Tyrosine | Dopamine precursor | Amino acid metabolism, other | amino acid related metabolism |
| Val | Valine | Valine metabolism | BCAA metabolism | amino acid related metabolism |
| C0 | Carnitine free | Carnitine transport | Carnitine transport | carnitine-related metabolism |
| C10 | Decanoylcarnitine | Medium chain fatty acid metabolism | Fatty acid metabolism | carnitine-related metabolism |
| C10:1 | Decenoylcarnitine | Medium chain fatty acid metabolism | Fatty acid metabolism | carnitine-related metabolism |
| C12 | Dodecanoylcarnitine | Medium chain fatty acid metabolism | Fatty acid metabolism | carnitine-related metabolism |
| C14 | Myristoylcarnitine | Long chain fatty acid metabolism | Fatty acid metabolism | carnitine-related metabolism |
| C14:1 | Tetradecenoylcarnitine | Long chain fatty acid metabolism | Fatty acid metabolism | carnitine-related metabolism |
| C14OH | 3-Hydroxy-tetradecanoylcarnitine | Long chain fatty acid metabolism | Fatty acid metabolism | carnitine-related metabolism |
| C16 | Palmitoylcarnitine | Long chain fatty acid metabolism | Fatty acid metabolism | carnitine-related metabolism |
| C16:1 | Hexadecenoylcarnitine | Long chain fatty acid metabolism | Fatty acid metabolism | carnitine-related metabolism |
| C16:1OH | 3-Hydroxy-hexadecenoylcarnitine | Long chain fatty acid metabolism | Fatty acid metabolism | carnitine-related metabolism |
| C16OH | 3-Hydroxy-hexadecanoylcarnitine | Long chain fatty acid metabolism | Fatty acid metabolism | carnitine-related metabolism |
| C18 | Stearoylcarnitine | Long chain fatty acid metabolism | Fatty acid metabolism | carnitine-related metabolism |
| C18:1 | Octadecenoylcarnitine | Long chain fatty acid metabolism | Fatty acid metabolism | carnitine-related metabolism |
| C18:1OH | Hydroxy-octadec-1-enoylcarnitine | Long chain fatty acid metabolism | Fatty acid metabolism | carnitine-related metabolism |
| C18:2 | Trans, trans-9,12-octadecadienoic acid (Linoelaidic) | Long chain fatty acid metabolism | Fatty acid metabolism | carnitine-related metabolism |
| C18:2OH | Hydroxy-octadec-2-enoylcarnitine | Long chain fatty acid metabolism | Fatty acid metabolism | carnitine-related metabolism |
| C18OH | 3-Hydroxy-octadecanoylcarnitine | Long chain fatty acid metabolism | Fatty acid metabolism | carnitine-related metabolism |
| C2 | Acetylcarnitine | Energy metabolism | Energy metabolism | carnitine-related metabolism |
| C20:1 | Cis-11-eicosenoic acid | PUFA metabolism | Fatty acid metabolism | carnitine-related metabolism |
| C20:2 | Cis-11,14-eicosadienoic acid | PUFA metabolism | Fatty acid metabolism | carnitine-related metabolism |
| C20:3 | Cis-11,14,17-eicosatrienoic acid methyl ester | PUFA metabolism | Fatty acid metabolism | carnitine-related metabolism |
| C3 | Propionylcarnitine | Isoleucine metabolism, Valine metabolism | BCAA metabolism | carnitine-related metabolism |
| C3DC | Malonylcarnitine | Energy metabolism, Fatty acid synthesis | BCAA metabolism | carnitine-related metabolism |
| C4 | Butyrylcarnitine | Short chain fatty acid metabolism | Energy metabolism | carnitine-related metabolism |
| C4OH | 3-Hydroxy-butyryl-carnitine | Energy metabolism | Energy metabolism | carnitine-related metabolism |
| C5 | Isovalerylcarnitine | Leucine metabolism | BCAA metabolism | carnitine-related metabolism |
| C5:1 | Triglylcarnitine | Isoleucine metabolism, Valine metabolism | BCAA metabolism | carnitine-related metabolism |
| C5OH | 2-Hydroxyisovalerylcarnitine | Leucine metabolism | BCAA metabolism | carnitine-related metabolism |
| C6 | Hexanoylcarnitine | Medium chain fatty acid metabolism | Fatty acid metabolism | carnitine-related metabolism |
| C61DC | Adipylcarnitine | Keton body generation from beta oxidation and ketogenic amino acids | Fatty acid metabolism | carnitine-related metabolism |
| C8 | Octanoylcarnitine | Medium chain fatty acid metabolism | Fatty acid metabolism | carnitine-related metabolism |
| C8:1 | Octenoylcarnitine | Medium chain fatty acid metabolism | Fatty acid metabolism | carnitine-related metabolism |
| MeGlut | 3-Methylglutarylcarnitine | Leucine metabolism | Amino acid metabolism, other | carnitine-related metabolism |
| C18DC | Dicarboxystearoylcarnitine | Fatty Acid Oxidation | Lipid Metabolism | carnitine-related metabolism |
| C5DC | Glutarylcarntine | Amino Acid Metabolism | Energy Metabolism | carnitine-related metabolism |
| C4DC=MMA | Methylmalonylcarnitine | Propionate Metabolism | Energy Metabolism | carnitine-related metabolism |
| C24 | Lignoceroylcarnitine C24 | Very Long Chain Fatty Acid Oxidation | Lipid Metabolism | carnitine-related metabolism |
| C26 | Cerotoylcarnitine C26 | Very Long Chain Fatty Acid Oxidation | Lipid Metabolism | carnitine-related metabolism |
| C16DC | Dicarboxypalmitoylcarnitine | Fatty Acid Oxidation | Lipid Metabolism | carnitine-related metabolism |
| C16:1 | Palmitoleic acid | Monounsaturated Fatty Acid Metabolism | Lipid Metabolism | fatty acid-related metabolism |
| C18:1 n-9 | Oleic acid | Monounsaturated Fatty Acid Metabolism | Lipid Metabolism | fatty acid-related metabolism |
| C18:2 n-6 | Linoleic acid | Polyunsaturated Fatty Acid Metabolism | Lipid Metabolism | fatty acid-related metabolism |
| C18:3 n-3 | alpha-Linolenic acid | Polyunsaturated Fatty Acid Metabolism | Lipid Metabolism | fatty acid-related metabolism |
| *C18:4.n-3* | *Stearidonic acid* | *Polyunsaturated Fatty Acid Metabolism* | *Lipid Metabolism* | fatty acid-related metabolism |
| C20:3 n-6 | Dihomo-gamma-linolenic acid | Arachidonic Acid Metabolism | Lipid Metabolism | fatty acid-related metabolism |
| C20:4 n-6 | Arachidonic acid | Arachidonic Acid Metabolism | Lipid Metabolism | fatty acid-related metabolism |
| C20:5 n-3 | Eicosapentaenoic acid | Omega-3 Fatty Acid Metabolism | Lipid Metabolism | fatty acid-related metabolism |
| *C22:4.n-6* | *Adrenic acid* | *Arachidonic Acid Metabolism* | *Lipid Metabolism* | fatty acid-related metabolism |
| C22:5 n-3 | Docosapentaenoic acid (22n-3) | Omega-3 Fatty Acid Metabolism | Lipid Metabolism | fatty acid-related metabolism |
| C22:5.n.6 | Docosapentaenoic acid (22n-6) | Arachidonic Acid Metabolism | Lipid Metabolism | fatty acid-related metabolism |
| C22:6 n-3 | Docosahexaenoic acid | Omega-3 Fatty Acid Metabolism | Lipid Metabolism | fatty acid-related metabolism |
| Ratio C18:2 n-6 to C18:3 n-3 |  | Arachidonic Acid Metabolism | Lipid Metabolism | fatty acid-related metabolism |
| *TxB3* | *Thromboxan B3* | *Eicosanoid Synthesis* | *Lipid Metabolism* | eicosanoid-related metabolism |
| *PGD3* | *Prostaglandin D3* | *Eicosanoid Synthesis* | *Lipid Metabolism* | eicosanoid-related metabolism |
| *X6.keto.PGF1a* | *6-Keto Prostaglandin F1α* | *Eicosanoid Synthesis* | *Lipid Metabolism* | eicosanoid-related metabolism |
| *TxB2* | *Thromboxan B2* | *Eicosanoid Synthesis* | *Lipid Metabolism* | eicosanoid-related metabolism |
| *PGF1a* | *Prostaglandin F1α* | *Eicosanoid Synthesis* | *Lipid Metabolism* | eicosanoid-related metabolism |
| *11.dehydro.TxB2* | *11-Dehydro Thromboxan B2* | *Eicosanoid Synthesis* | *Lipid Metabolism* | eicosanoid-related metabolism |
| Tetranor.12.S..HETE | 8S-hydroxy-4Z,6E,10Z-hexadecatrienoic acid | Eicosanoid Synthesis | Lipid Metabolism | eicosanoid-related metabolism |
| *14.15.DHET* | *14,15-dihydroxy-5Z,8Z,11Z- (5Z,8Z,11Z) -14,15-dihydroxyeicosa-5,8,11-trienoic acid* | *Eicosanoid Synthesis* | *Lipid Metabolism* | eicosanoid-related metabolism |
| *12.S..HHT* | *12(S)-Hydroxyheptadecatrienoic acid* | *Eicosanoid Synthesis* | *Lipid Metabolism* | eicosanoid-related metabolism |
| *11.12.DHET* | *11,12-Dihydroxy-eicosatrienoic acid* | *Eicosanoid Synthesis* | *Lipid Metabolism* | eicosanoid-related metabolism |
| *5.6.DHET* | *5,6-Dihydroxy-eicosatrienoic acid* | *Eicosanoid Synthesis* | *Lipid Metabolism* | eicosanoid-related metabolism |
| *8.9.DHET* | *8,9-Dihydroxy-eicosatrienoic acid* | *Eicosanoid Synthesis* | *Lipid Metabolism* | eicosanoid-related metabolism |
| *18.HETE* | *18-Hydroxyeicosatetraenoic acid* | *Eicosanoid Synthesis* | *Lipid Metabolism* | eicosanoid-related metabolism |
| *12.S..HEPE* | *12(S)-Hydroxyeicosapentaenoic acid* | *Eicosanoid Synthesis* | *Lipid Metabolism* | eicosanoid-related metabolism |
| *17.S..HETE* | *17(S)-Hydroxyeicosatetraenoic acid* | *Eicosanoid Synthesis* | *Lipid Metabolism* | eicosanoid-related metabolism |
| *16.S..HETE* | *16(S)-Hydroxyeicosatetraenoic acid* | *Eicosanoid Synthesis* | *Lipid Metabolism* | eicosanoid-related metabolism |
| *5.S..HEPE* | *5(S)-Hydroxyeicosapentaenoic acid* | *Eicosanoid Synthesis* | *Lipid Metabolism* | eicosanoid-related metabolism |
| 13.HODE | 13-Hydroxyoctadecadienoic acid | Linoleic Acid Metabolism | Lipid Metabolism | eicosanoid-related metabolism |
| 9.HODE | 9(S)-Hydroxyoctadecadienoic acid (alpha-Dimorphecolic acid) | Linoleic Acid Metabolism | Lipid Metabolism | eicosanoid-related metabolism |
| *15.S..HETE* | *15(S)-Hydroxyeicosatetraenoic acid* | *Eicosanoid Synthesis* | *Lipid Metabolism* | eicosanoid-related metabolism |
| *11.S..HETE* | *11(S)-Hydroxyeicosatetraenoic acid* | *Eicosanoid Synthesis* | *Lipid Metabolism* | eicosanoid-related metabolism |
| *8.S..HETE.12.HETE* | *8(S)-Hydroxyeicosatetraenoic acid / 12-Hydroxyeicosatetraenoic acid* | *Eicosanoid Synthesis* | *Lipid Metabolism* | eicosanoid-related metabolism |
| *5.S..HETE* | *5(S)-Hydroxyeicosatetraenoic acid* | *Eicosanoid Synthesis* | *Lipid Metabolism* | eicosanoid-related metabolism |
| *12.oxo.ETE* | *12-Oxo-eicosatetraenoic acid* | *Eicosanoid Synthesis* | *Lipid Metabolism* | eicosanoid-related metabolism |
| *Resolvin..E1* | *Resolvin E1* | *Specialized Pro-resolving Mediators* | *Lipid Metabolism* | resolvin-related metabolism |
| 17.18.DiHETE | 17,18-Dihydroxyeicosatetraenoic acid | Specialized Pro-resolving Mediators | Lipid Metabolism | resolvin-related metabolism |
| *14.15.DiHETE* | *14,15-Dihydroxyeicosatetraenoic acid* | *Specialized Pro-resolving Mediators* | *Lipid Metabolism* | resolvin-related metabolism |
| 19.20.DiHDPA | 19,20-Dihydroxydocosapentaenoic acid | Specialized Pro-resolving Mediators | Lipid Metabolism | resolvin-related metabolism |
| *18.HEPE* | *18-Hydroxyeicosapentaenoic acid* | *Specialized Pro-resolving Mediators* | *Lipid Metabolism* | resolvin-related metabolism |
| *8.HEPE* | *8-Hydroxyeicosapentaenoic acid* | *Specialized Pro-resolving Mediators* | *Lipid Metabolism* | resolvin-related metabolism |
| *20.HDoHE* | *20-Hydroxydocosahexaenoic acid* | *Specialized Pro-resolving Mediators* | *Lipid Metabolism* | resolvin-related metabolism |
| *16.HDoHE* | *16-Hydroxydocosahexaenoic acid* | *Specialized Pro-resolving Mediators* | *Lipid Metabolism* | resolvin-related metabolism |
| *10.HDoHE* | *10-Hydroxydocosahexaenoic acid* | *Specialized Pro-resolving Mediators* | *Lipid Metabolism* | resolvin-related metabolism |
| *14.S..HDoHE* | *14(S)-Hydroxydocosahexaenoic acid* | *Specialized Pro-resolving Mediators* | *Lipid Metabolism* | resolvin-related metabolism |
| *19.20..EpDPE* | *19,20-Epoxydocosapentaenoic acid* | *Specialized Pro-resolving Mediators* | *Lipid Metabolism* | resolvin-related metabolism |
